# Supplementary material for: PenDA, a rank-based method for personalized differential analysis: Application to lung cancer
Source: PLoS Comput Biol. 2020 May 11;16(5):e1007869. doi: 10.1371/journal.pcbi.1007869 (PMC7274464; doi:10.1371/journal.pcbi.1007869)
Supplement: S1 Fig — We plotted the evolution of the total number of predicted deregulated genes during the successive iterations of the PenDA method applied to one simulated dataset with l = 30 and h = 0.1 (red line) or h = 0.4 (cyan line). (PDF) [file pcbi.1007869.s001.pdf]

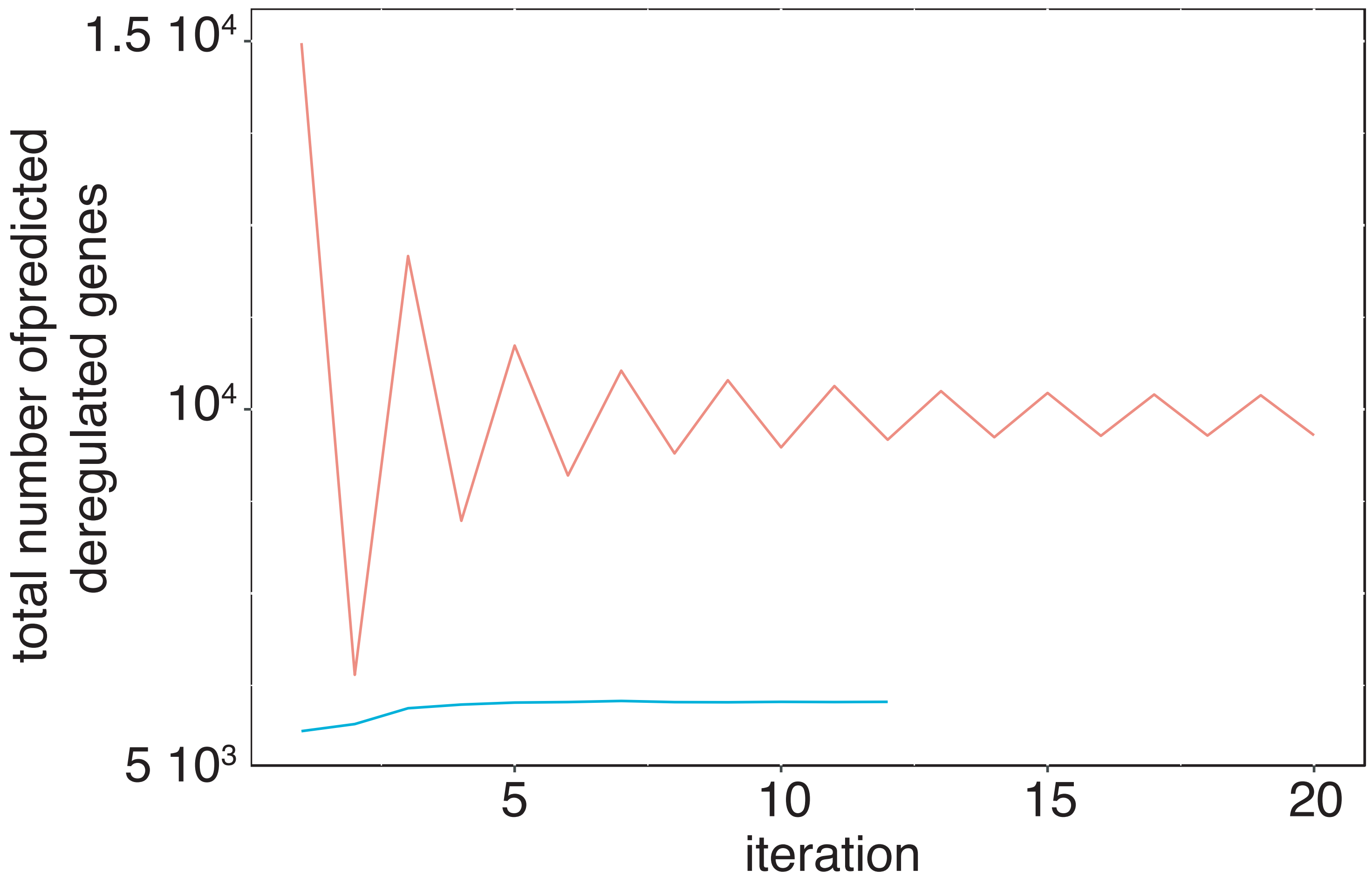

S1 Fig. Convergence towards a consistent list of deregulated genes is rapidly achieved by the PenDA method. We plotted the evolution of the total number of predicted deregulated genes during the successive iterations of the PenDA method applied to one simulated dataset with l=30 and h=0.1 (red line) or h=0.4 (cyan line).
