# Supplementary material for: PenDA, a rank-based method for personalized differential analysis: Application to lung cancer
Source: PLoS Comput Biol. 2020 May 11;16(5):e1007869. doi: 10.1371/journal.pcbi.1007869 (PMC7274464; doi:10.1371/journal.pcbi.1007869)
Supplement: S2 Fig — (a) ROC curve of the percentile method obtained by varying parameter x for different values of factor f. TPR and FPR were computed on a set of 10 simulations. (b) Maximal informedness of the ROC curve as a function of f. (PDF) [file pcbi.1007869.s002.pdf]

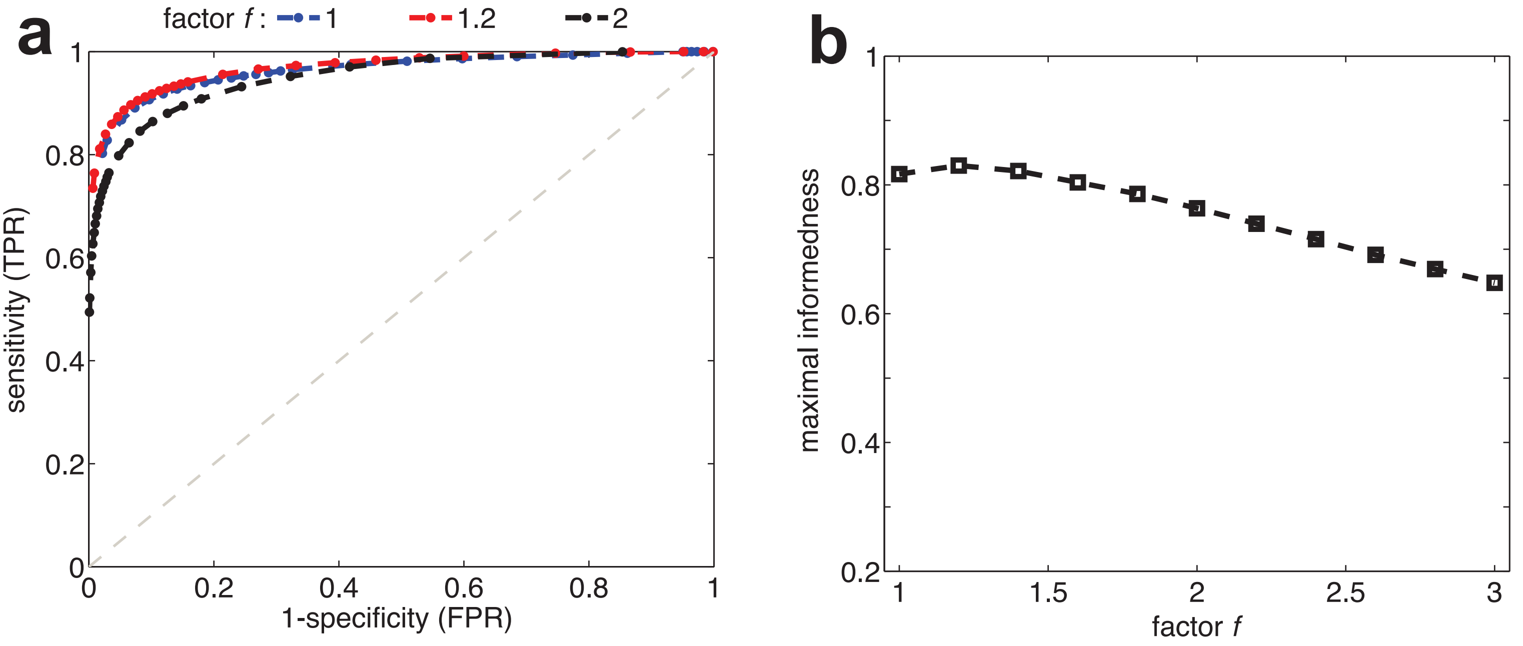

S2 Fig. Test of the percentile method. (a) ROC curve of the percentile method obtained by varying parameter x for different values of factor f. TPR and FPR were computed on a set of 10 simulations. (b) Maximal informedness of the ROC curve as a function of f.
