# Supplementary figures and images for: PenDA, a rank-based method for personalized differential analysis: Application to lung cancer
Source: PLoS Comput Biol. 2020 May 11;16(5):e1007869. doi: 10.1371/journal.pcbi.1007869 (PMC7274464; doi:10.1371/journal.pcbi.1007869)

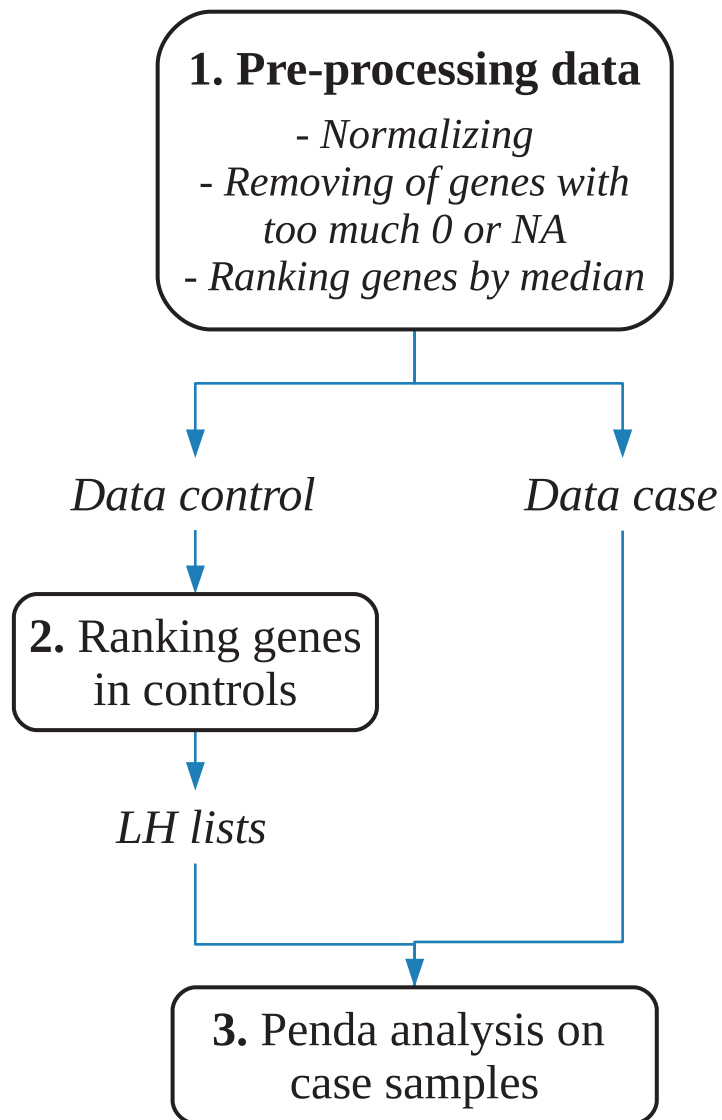

**S3 Fig.** PenDA workflow.

Supplement: S3 Fig — (PDF) [file pcbi.1007869.s003.pdf]
