# Supplementary material for: PenDA, a rank-based method for personalized differential analysis: Application to lung cancer
Source: PLoS Comput Biol. 2020 May 11;16(5):e1007869. doi: 10.1371/journal.pcbi.1007869 (PMC7274464; doi:10.1371/journal.pcbi.1007869)
Supplement: S4 Fig — (a,b) Effect of tumor stages on the total number of gene deregulations in both ADC (a) and SQCC patients (b). (c,d) Effect of tumor stages on down/up ratios in both ADC (c) and SQCC patients (d). Significance was assessed via one-way ANOVA with Tukey’s multiple comparison post hoc test, considering the stage as an independent factor, with 5 different levels (stage ia, stage ib, stage ii, stage iii and stage iv). LUAD gene deregulation: Df = 4, F-statistic = 5.18, p-value = 0.0004. LUAD deregulation ratio: Df = 4, F-statistic = 0.99, p-value = 0.4128. LUSC gene deregulation: Df = 4, F-statistic = 3.00, p-value = 0.0182. LUAD deregulation ratio: Df = 4, F-statistic = 1.59, p-value = 0.1767. Dashed red lines represent 1st quartile, median and 3rd quartile of the distributions. (PDF) [file pcbi.1007869.s004.pdf]

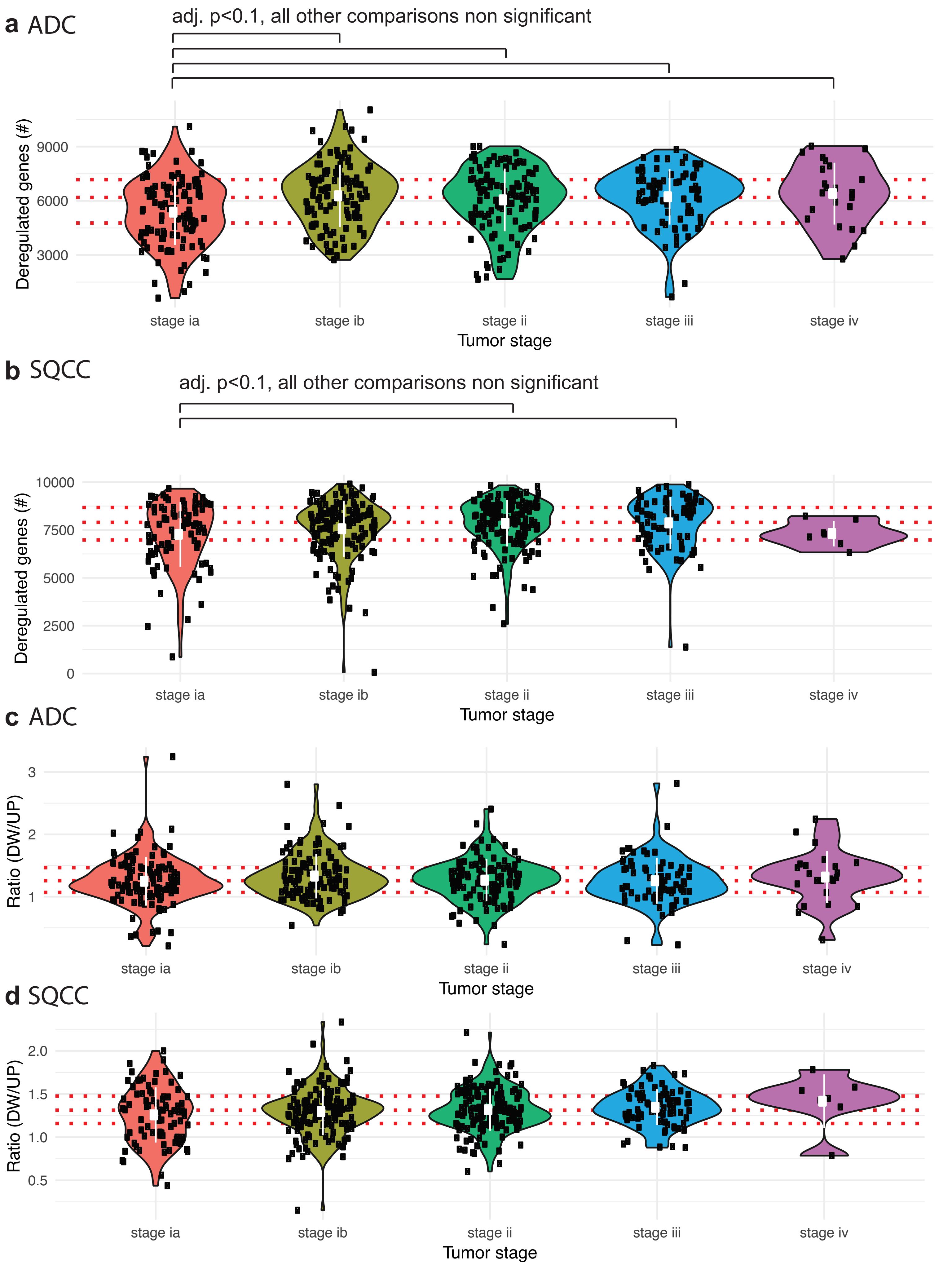

S4 Fig. Effect of tumor stages on the total number of gene deregulations in both ADC (a) and SQCC patients (b). Effect of tumor stages on down/up ratios in both ADC (c) and SQCC patients (d). Significance was assessed via one-way ANOVA with Tukey's multiple comparison post hoc test, considering the stage as an independent factor, with 5 different levels (stage ia, stage ib, stage ii, stage iii and stage iv). LUAD gene deregulation: Df = 4, F-statistic = 5.18, p-value = 0.0004. LUAD deregulation ratio: Df = 4, F-statistic = 0.99, p-value = 0.4128. LUSC gene deregulation: Df = 4, F-statistic = 3.00, p-value = 0.0182. LUAD deregulation ratio: Df = 4, F-statistic = 1.59, p-value = 0.1767. Dashed red lines represent 1st quartile, median and 3rd quartile of the distributions.
