# Supplementary material for: PenDA, a rank-based method for personalized differential analysis: Application to lung cancer
Source: PLoS Comput Biol. 2020 May 11;16(5):e1007869. doi: 10.1371/journal.pcbi.1007869 (PMC7274464; doi:10.1371/journal.pcbi.1007869)
Supplement: S6 Fig — Genes deregulated in more than 30% of the patient are depicted in the diagram. x-axis corresponds to the % of up-regulation/total-deregulation in ADC, y-axis corresponds to the % of up-regulation/ total-deregulation in SQCC. Each dot (gray cross) corresponds to one gene. Blue points correspond to super-down-regulated genes, red points correspond to super-up-regulated genes (triangles for ADC, circles for SQCC). Diamonds black points represent genes displaying antagonistic commitment behaviour between ADC and SQCC (~5% of the total number of genes depicted). (PDF) [file pcbi.1007869.s006.pdf]

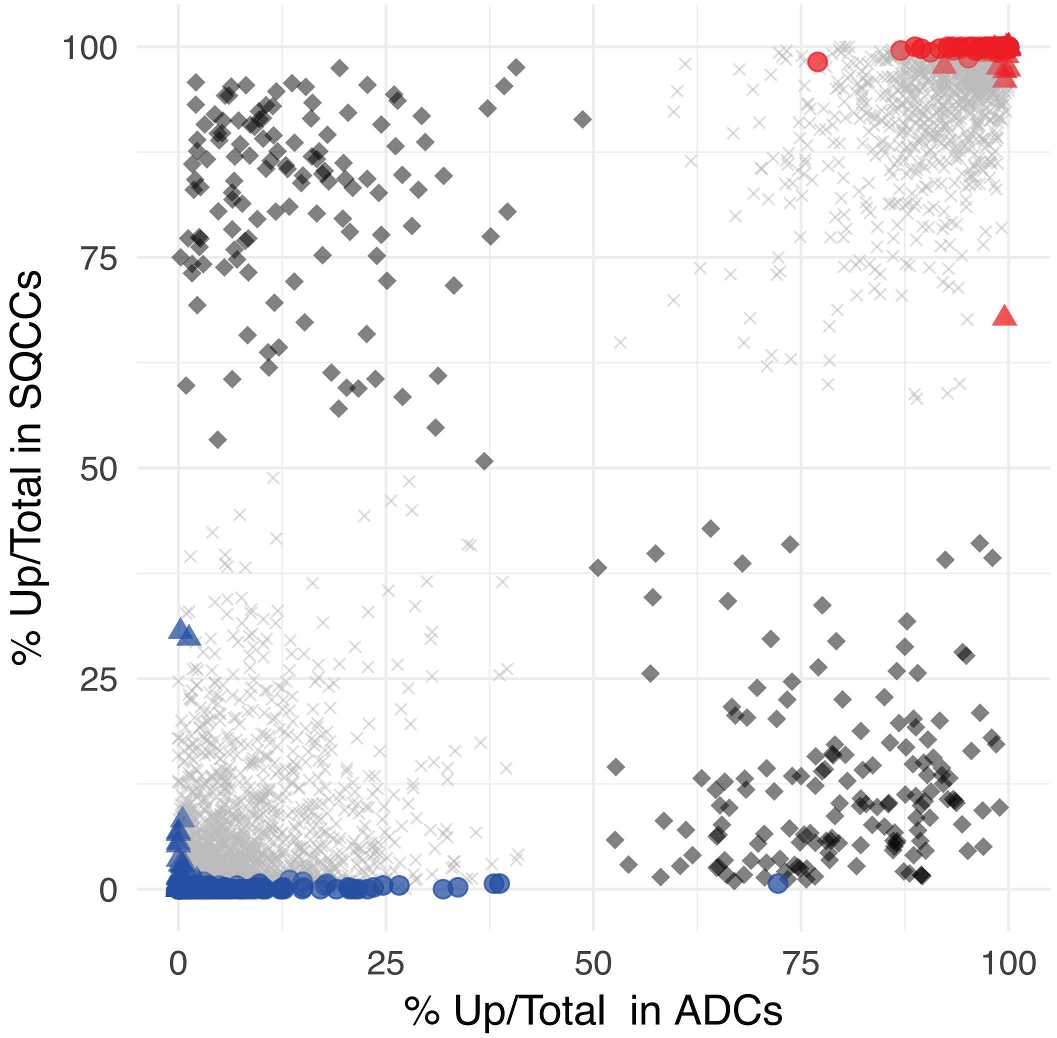

S6 Fig. Gene deregulation commitment in ADCs and SQCCs. Genes deregulated in more than 30 % of the patient are depicted in the diagram. x-axis corresponds to the % of up-regulation/total-deregulation in ADC, y-axis corresponds to the % of up-regulation/-total-deregulation in SQCC. Each dot (gray cross) corresponds to one gene. Blue points correspond to super-down-regulated genes, red points correspond to super-up-regulated genes (triangles for ADC, circles for SQCC). Diamonds black points represent genes displaying antagonistic commitment behaviour between ADC and SQCC (~5% of the total number of genes depicted).
