# Supplementary material for: PenDA, a rank-based method for personalized differential analysis: Application to lung cancer
Source: PLoS Comput Biol. 2020 May 11;16(5):e1007869. doi: 10.1371/journal.pcbi.1007869 (PMC7274464; doi:10.1371/journal.pcbi.1007869)
Supplement: S7 Fig — GO (biological pathways) analysis for super-up-regulated (a) and super-down-regulated (b) genes in ADC or SQCC. Rows of the heatmap correspond to genes overlapping with at least one enriched term (red). Genes with no overlapping terms were removed from the graphical representation. Columns correspond to enriched terms clustered by Euclidean distance. GO Term significance score corresponds to -log10 of the Modified Fisher Exact P-Value after Benjamini correction (extracted from DAVID’s Functional Annotation tool). (PDF) [file pcbi.1007869.s007.pdf]

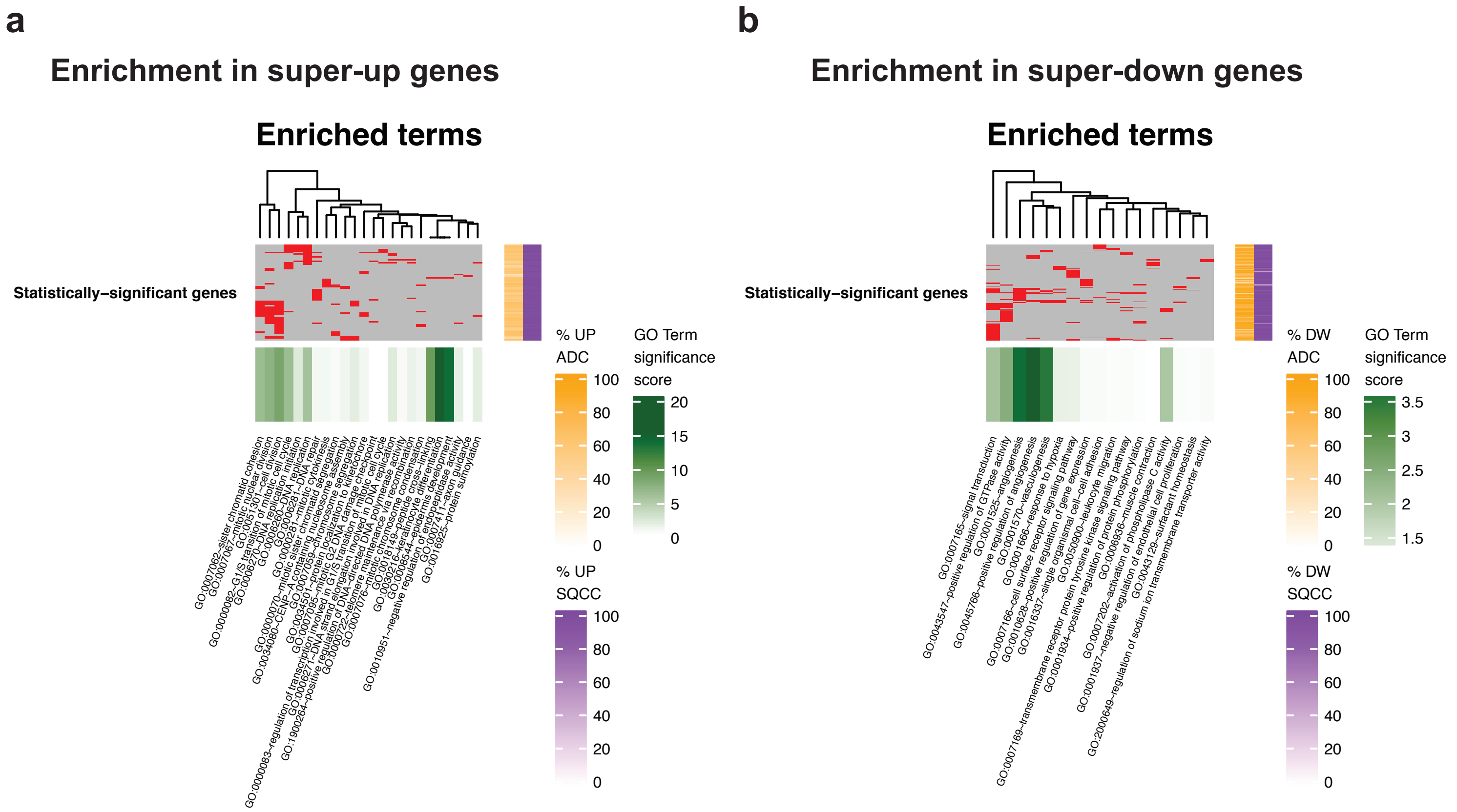

S7 Fig. Gene Ontology (biological pathways) enrichment in genes super-up-regulated in ADC or SQCC (a) and genes super-down-regulated in ADC or SQCC (b). Rows of the heatmap correspond to genes overlapping with at least one enriched term (red). Genes with no overlapping terms were removed from the graphical representation. Columns correspond to enriched terms clustered by Euclidean distance. GO Term significance score corresponds to -log10 of the Modified Fisher Exact P-Value after Benjamini correction (extracted from DAVID's Functional Annotation tool).
