# Supplementary material for: PenDA, a rank-based method for personalized differential analysis: Application to lung cancer
Source: PLoS Comput Biol. 2020 May 11;16(5):e1007869. doi: 10.1371/journal.pcbi.1007869 (PMC7274464; doi:10.1371/journal.pcbi.1007869)
Supplement: S8 Fig — (a) Graphical representation of the contingency table between ADC iCluster (The Cancer Genome Atlas Research Network) and ADC subclasses (PenDA analysis). (b) Heatmap of normalized counts matrix applied to a specific set of classifier genes (n = 875) in TCGA non-small-cell lung cancers: ADC (orange) and SQCC (purple). Two hierarchical clusterings were performed: using Euclidean distance to sort genes and using Pearson correlation-based distance to classify patients, with a complete linkage function in both cases. ADC subclasses defined by PenDA analysis (colour-coded, class I to III) are defined according to Fig 6 of the main text. (PDF) [file pcbi.1007869.s008.pdf]

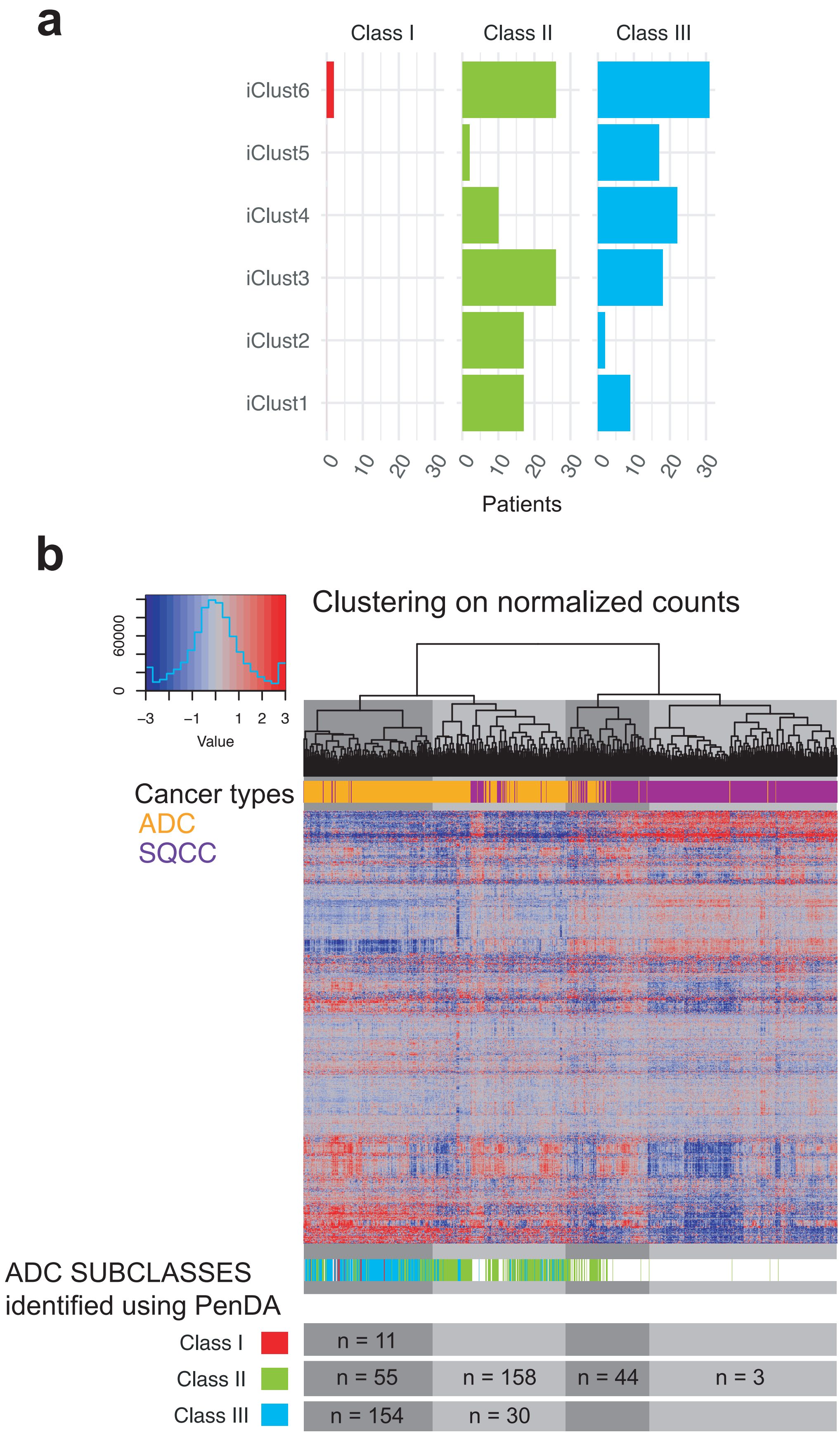

S8 Fig. (a) Graphical representation of the contingency table between ADC iCluster (The Cancer Genome Atlas Research Network) and ADC subclasses (PenDA analysis). (b) Heatmap of normalized counts matrix applied to a specific set of classifier genes (n = 875) in TCGA non-small-cell lung cancers: ADC (orange) and SQCC (purple). Two hierarchical clusterings were performed: using Euclidean distance to sort genes and using Pearson correlation based distance to classify patients, with a complete linkage function in both cases. ADC subclasses defined by PenDA analysis (colour-coded, class I to III) are defined according to figure 6.
