# Supplementary material for: PenDA, a rank-based method for personalized differential analysis: Application to lung cancer
Source: PLoS Comput Biol. 2020 May 11;16(5):e1007869. doi: 10.1371/journal.pcbi.1007869 (PMC7274464; doi:10.1371/journal.pcbi.1007869)
Supplement: S9 Fig — (a) Effect of cancer stage patients (chi-square test p-value = 0.2133). (b) Effect of gender (chi square test p-value = 1). (c) Effect of age patients (chi square test p-value = 0.2133). (PDF) [file pcbi.1007869.s009.pdf]

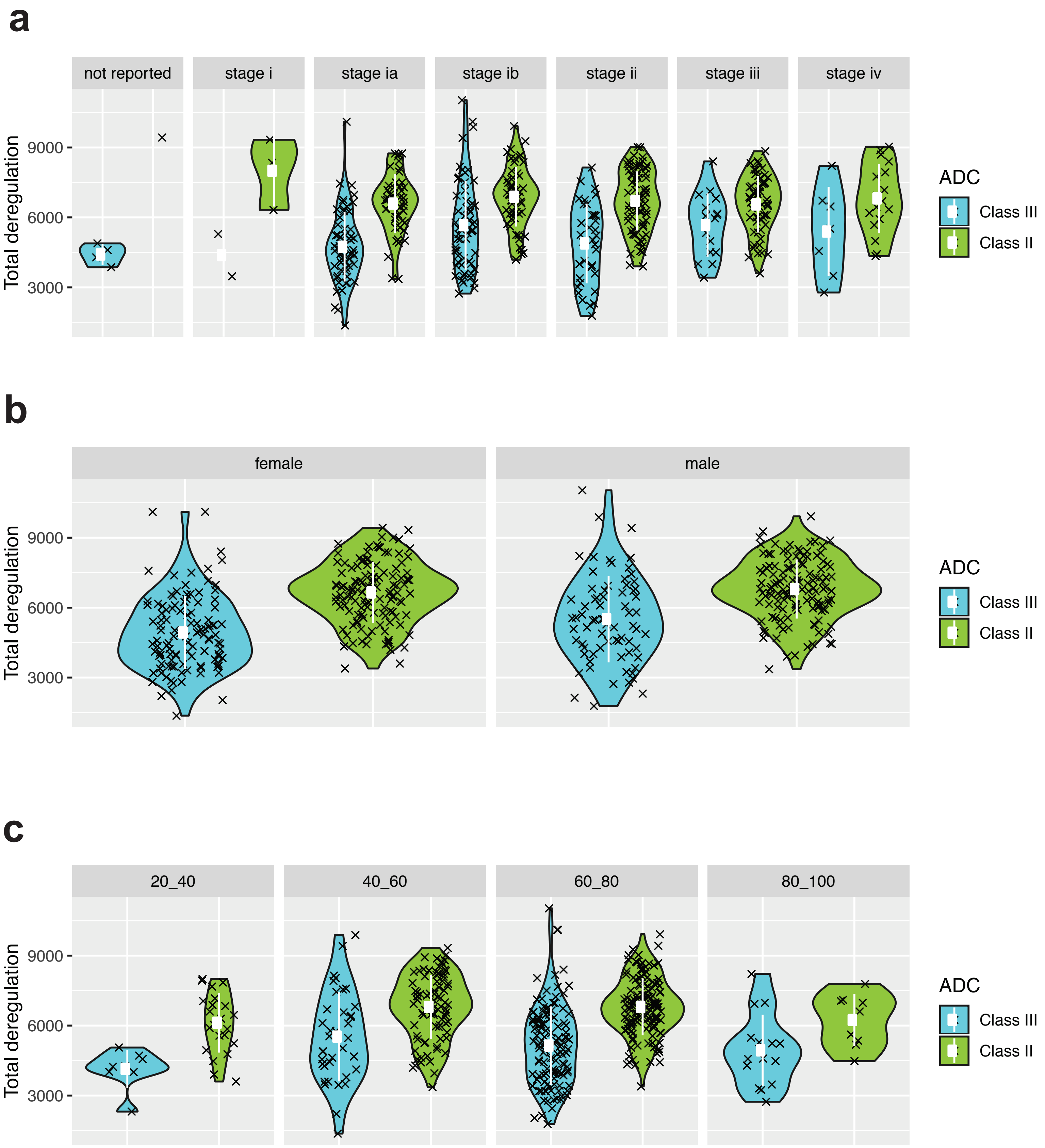

S9 Fig. Effect of putative confounding factors on ADC classification in class II and III. (a) Effect of cancer stage patients (chi square test p-value = 0.2133). (b) Effect of gender (chi square test p-value = 1). (c) Effect of age patients (chi square test p-value = 0.2133).
