# Supplementary material for: PenDA, a rank-based method for personalized differential analysis: Application to lung cancer
Source: PLoS Comput Biol. 2020 May 11;16(5):e1007869. doi: 10.1371/journal.pcbi.1007869 (PMC7274464; doi:10.1371/journal.pcbi.1007869)
Supplement: S10 Fig — DESeq2 analysis of the ADC (a) and SQCC (b) TCGA cohorts (green triangles: super-conserved genes, red triangles: super-up-regulated genes, blue triangles: super-down-regulated genes). (c) Genes identified as deregulated by Tian et al. x-axis corresponds to normalized mean expression in controls. y-axis corresponds to normalized mean expression in tumor. Gene with super patterns identified with PenDA are depicted with triangles (green triangles: super-conserved genes, red triangles: super-up-regulated genes, blue triangles: super-down-regulated genes). (PDF) [file pcbi.1007869.s010.pdf]

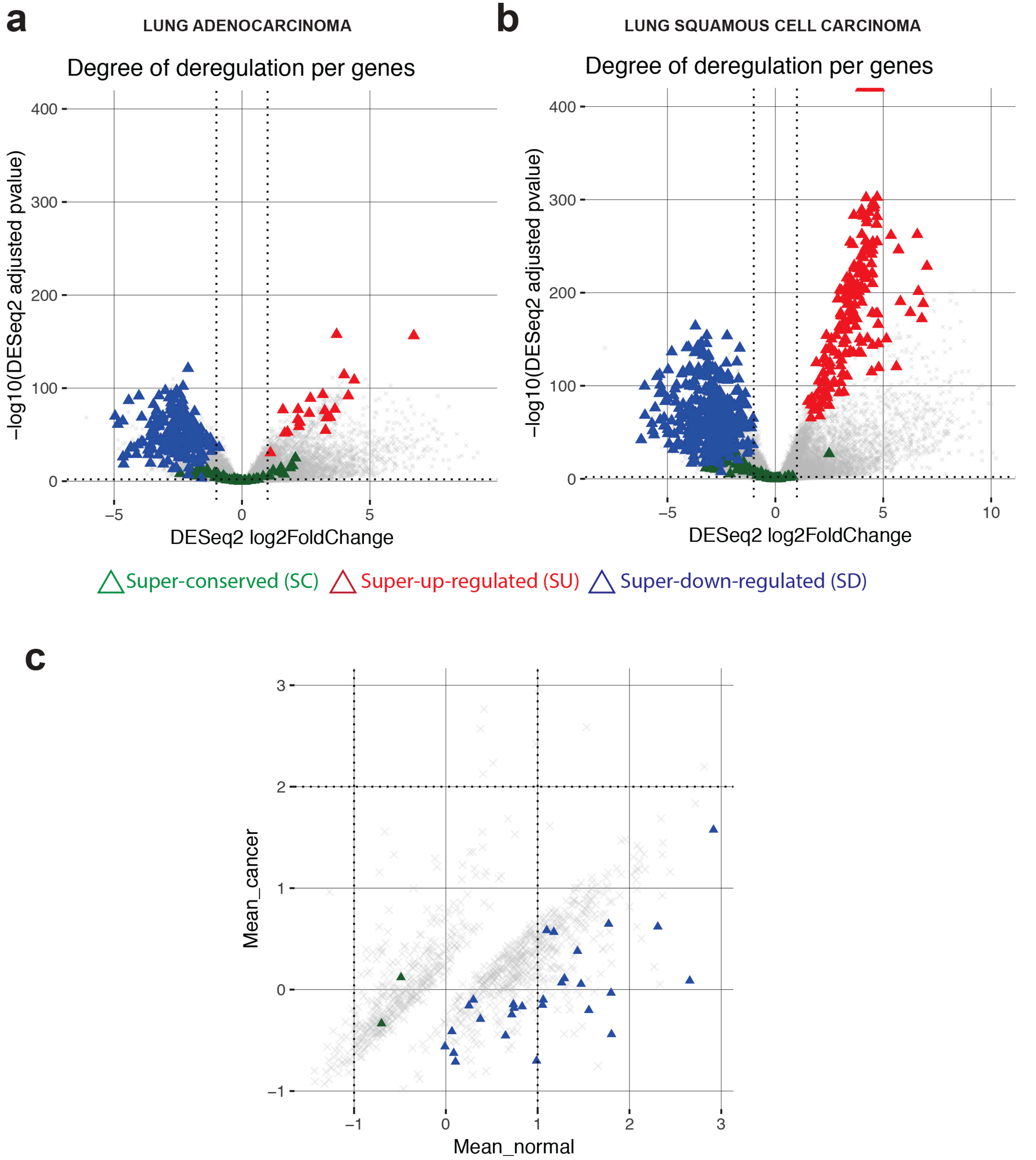

S10 Fig. DESeq2 analysis of the ADC (a) and SQCC (b) TCGA cohorts (green triangles: super-conserved genes, red triangles: super-up-regulated genes, blue triangles: super-down-regulated genes). (c) Genes identified as deregulated by Tian et al. x-axis corresponds to normalized mean expression in controls. y-axis corresponds to normalized mean expression in tumor. Gene with super patterns identified with PenDA are depicted with triangles (green triangles: super-conserved genes, red triangles: super-up-regulated genes, blue triangles: super-down-regulated genes).
