# Supplementary material for: PenDA, a rank-based method for personalized differential analysis: Application to lung cancer
Source: PLoS Comput Biol. 2020 May 11;16(5):e1007869. doi: 10.1371/journal.pcbi.1007869 (PMC7274464; doi:10.1371/journal.pcbi.1007869)
Supplement: S11 Fig — (a) ROC curves (true positive rate TPR vs false positive rate FPR) of the PenDA method on different simulated datasets obtained with different packet sizes. The curves were obtained by varying the proportion threshold h. (b) Comparison with other methods. ROC curves on simulated datasets generated using percentiles 10%,90% (Left) and 20%,80% (Right) in the simulation method (see Methods in the main text) for PenDA, a simple percentile-based method, 2 versions of Rankcomp and DESeq2. (c) ROC curves of the full DESeq2 method (full lines) or with the DESeq2 method skipping the internal routine for normalization (dashed lines) for the same simulated dataset used in Fig 2 and Fig 3A of the main text (green) or with the non-standardized dataset used in Fig 3C of the main text. (PDF) [file pcbi.1007869.s011.pdf]

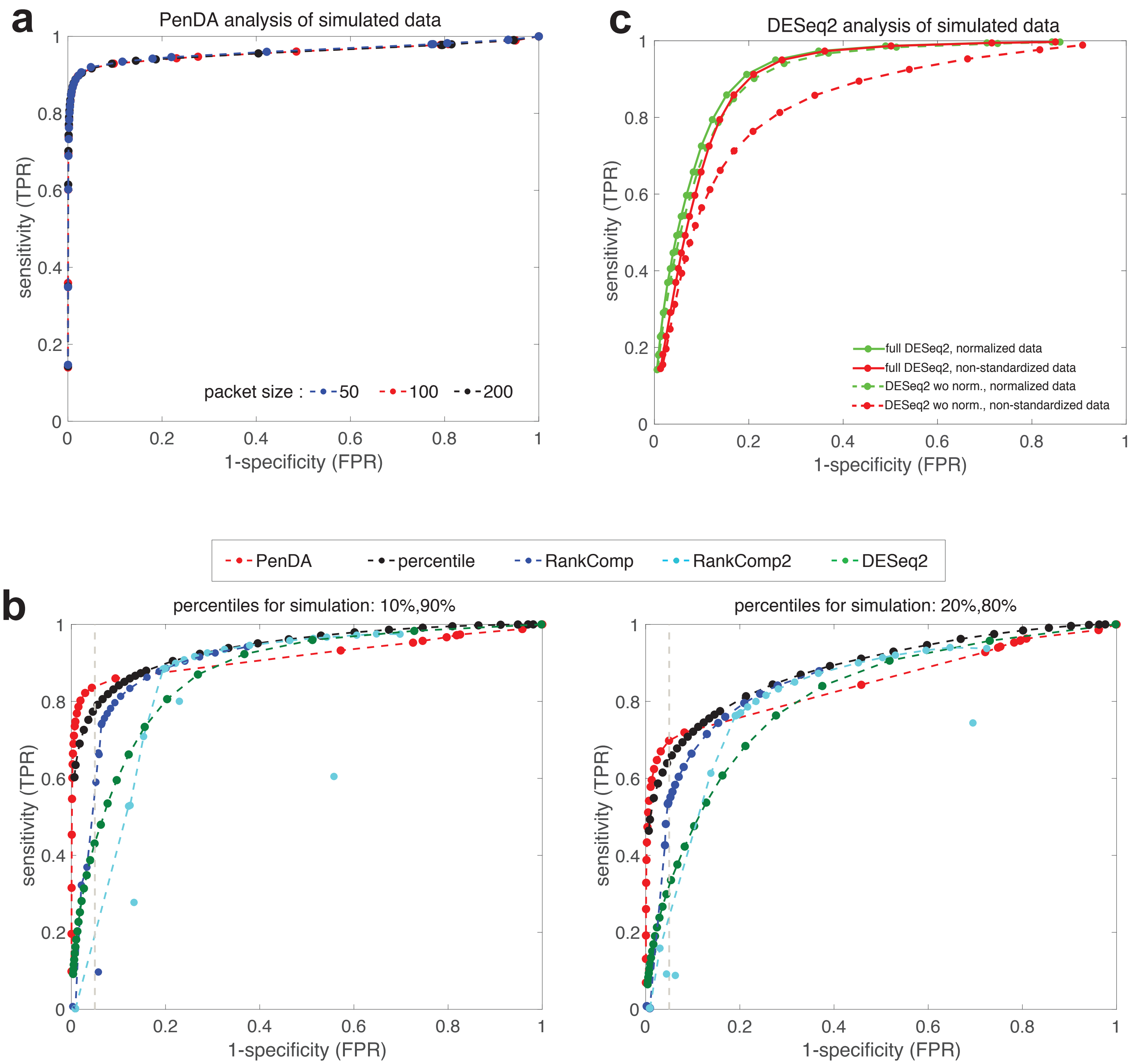


**S11 Fig.** (a) ROC curves (true positive rate TPR vs false positive rate FPR) of the PenDA method on different simulated datasets obtained with different packet sizes. The curves were obtained by varying the proportion threshold *h*. (b) Comparison with other methods. ROC curves on simulated datasets generated using percentiles 10%,90% (Left) and 20%,80% (Right) in the simulation method (see Methods in the main text) for PenDA, a simple percentile-based method, 2 versions of Rankcomp and DESeq2. (c) ROC curves of the full DESeq2 method (full lines) or with the DESeq2 method skipping the internal routine for normalization (dashed lines) for the same simulated dataset used in Fig.2 and Fig.3a of the main text (green) or with the non-standardized dataset used in Fig.3c of the main text.
