# Supplementary material for: PenDA, a rank-based method for personalized differential analysis: Application to lung cancer
Source: PLoS Comput Biol. 2020 May 11;16(5):e1007869. doi: 10.1371/journal.pcbi.1007869 (PMC7274464; doi:10.1371/journal.pcbi.1007869)
Supplement: S1 Text — (PDF) [file pcbi.1007869.s012.pdf]

PENDA: PErsoNalized Differential Analysis

Performing personalized data analysis with `penda`

Magali Richard, Clementine Decamps, Florent Chuffart, Daniel Jost

2019-06-06

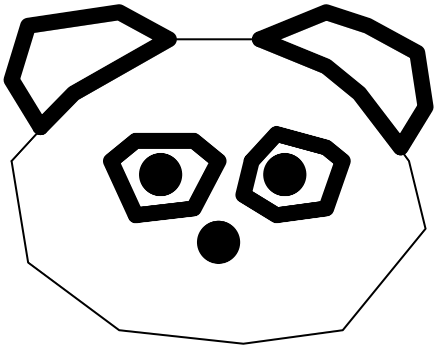

Introduction

`penda` (**PE**rsoNalized **D**ifferential **A**nalysis) is an open-access R package that detects gene deregulation in individual samples compared to a set of reference, control samples. This tutorial aims at providing to non-expert users basic informations and illustrations on how to run the package.

How to cite: Richard et al. (2019) PenDA, a rank-based method for Personalized Differential Analysis: application to lung cancer, in submission.

Dataset and data filtering

Dataset

The dataset used to illustrated the method corresponds to the transcriptomes of 3000 genes (RNAseq counts, normalized with DESeq2) for 40 normal, control samples and 40 tumorous samples taken from the TCGA study of lung adenocarcinoma [PMID:25079552].

`data_ctrl` is a data matrix containing the normalized counts of each control sample. The rownames of the matrix correspond to the gene_symbol, the colnames indicate the sample ID.

```
data_ctrl = penda::penda_data_ctrl
head(data_ctrl[,1:3])
#>       patient_55-6984-11 patient_43-6773-11 patient_55-6978-11
#> AADAC           347.2489           428.5498           442.0555
#> AAMP            965.2342          1528.3221           968.0266
#> ABCA1             0.0000             0.0000             0.0000
#> ABL1           1508.1784          1227.1325          1747.2431
#> ABL2            582.6719           645.4063           488.5088
#> ACACA             0.0000             0.0000             0.0000
dim(data_ctrl)
#> [1] 3000   40
```

`data_case` is a data matrix containing the normalized counts of each tumor sample. The rownames of the matrix correspond to the gene_symbol, the colnames indicate the sample ID.

```
data_case = penda::penda_data_case
data_case = data_case[rownames(data_ctrl),]
head(data_case[,1:3])
#>       patient_69-7764-01 patient_44-3919-01 patient_86-8278-01
#> AADAC           311.2129           374.9473          445.43169
#> AAMP           1466.5906           979.2256         1059.19225
#> ABCA1             0.0000             0.0000            0.00000
#> ABL1           2676.4306          2065.7474         2503.76905
#> ABL2           1167.0482           678.5603         1263.94317
#> ACACA             0.0000             0.0000           12.79693
dim(data_case)
#> [1] 3000   40
```

Note: this vignette is an example that has been designed for a rapid test of the method. So we limit the number of genes and the number of samples for this purpose. For an optimal utilization of the method, users should however upload all their available data (genes, control and case samples).

Method

`penda` performs a 3-steps analysis:

1. Data filtering and creation of the dataset
2. Relative gene ordering
3. Differential expression testing

Data filtering

```
threshold_dataset = 0.99
Penda_dataset = penda::make_dataset(data_ctrl, data_case, detectlowvalue = TRUE,
    detectNA = TRUE, threshold = threshold_dataset)
#> [1] "0 probes are NA in at least 99 % of the samples."
#> [1] "0 patients have NA for at least 99 % of the probes."
#> [1] "Computing of the low threshold"
#> number of iterations= 182
#> [1] "559 genes have less than 483.918718057525 counts in 99 % of the samples."
data_ctrl = Penda_dataset$data_ctrl
data_case = Penda_dataset$data_case
```

The function `make_dataset` contains three steps to prepare the data for the analysis.

- `detect_na_value` removes rows and columns (ie, genes and samples) of the data matrices that contain more than threshold % (default value = 0.99) of NA (Not Available) value.
- `detect_zero_value` removes genes with very low expression in the majority of samples (controls and cases), *ie.* genes whose expression is lower than `val_min` in `threshold`% of all the samples. By default it uses the function `normalmixEM` to estimate the value of `val_min` using all the *log2*-transformed count data but this parameter can also be tuned manually by the user.
- `rank_genes` sorts the genes based on the median value of gene expression in controls. This step is essential for the proper functioning of `penda`.

```r
head(data_ctrl[,1:3])
#>        patient_55-6984-11 patient_43-6773-11 patient_55-6978-11
#> GRIA1           0.0000000           0.000000           0.000000
#> POU3F4          0.0000000           1.721083           2.996986
#> KLF10           0.7356968           0.000000           0.000000
#> SPOP            0.7356968          13.768668           4.495480
#> PRMT3           0.0000000           0.000000           0.000000
#> KLF2            0.0000000           0.000000           0.000000
dim(data_ctrl)
#> [1] 2441   40
head(data_case[,1:3])
#>        patient_69-7764-01 patient_44-3919-01 patient_86-8278-01
#> GRIA1             0.00000          0.5895398           0.000000
#> POU3F4            0.00000          0.0000000           0.000000
#> KLF10             0.00000          0.5895398           0.000000
#> SPOP           1989.16884          0.0000000         169.805449
#> PRMT3            85.58354         88.4309664         324.845208
#> KLF2              0.00000         38.3200855           7.382846
dim(data_case)
#> [1] 2441   40
```

Relative gene ordering

```r
threshold_LH = 0.99
s_max = 30
L_H_list = penda::compute_lower_and_higher_lists(data_ctrl, threshold = threshold_LH,
    s_max = s_max)
#> [1] "Computing genes with lower and higher expression"
L = L_H_list$L
H = L_H_list$H
```

The `penda` method uses the relative gene ordering in normal tissue.

The function `compute_lower_and_higher_lists` computes two matrices `L` and `H` based on the filtered control dataset (`data_ctrl`).

Each row of the **L** matrix contains a list of at most `s_max` (default value = 30) genes (characterized by their ids) whose expressions are **lower** than that of the gene associated to the corresponding row, in at least `threshold_LH` (default value = 99 %) of the control samples.

Each row of the **H** matrix contains a list of at most `s_max` (default value = 30) genes (characterized by their ids) whose expressions are **higher** than that of the gene associated to the corresponding row, in at least `threshold_LH` (default value = 99 %) of the control samples.

Below, for some genes (FOXH1, KRTAP2-3, etc.), we show the id of 10 genes of the L and H lists.

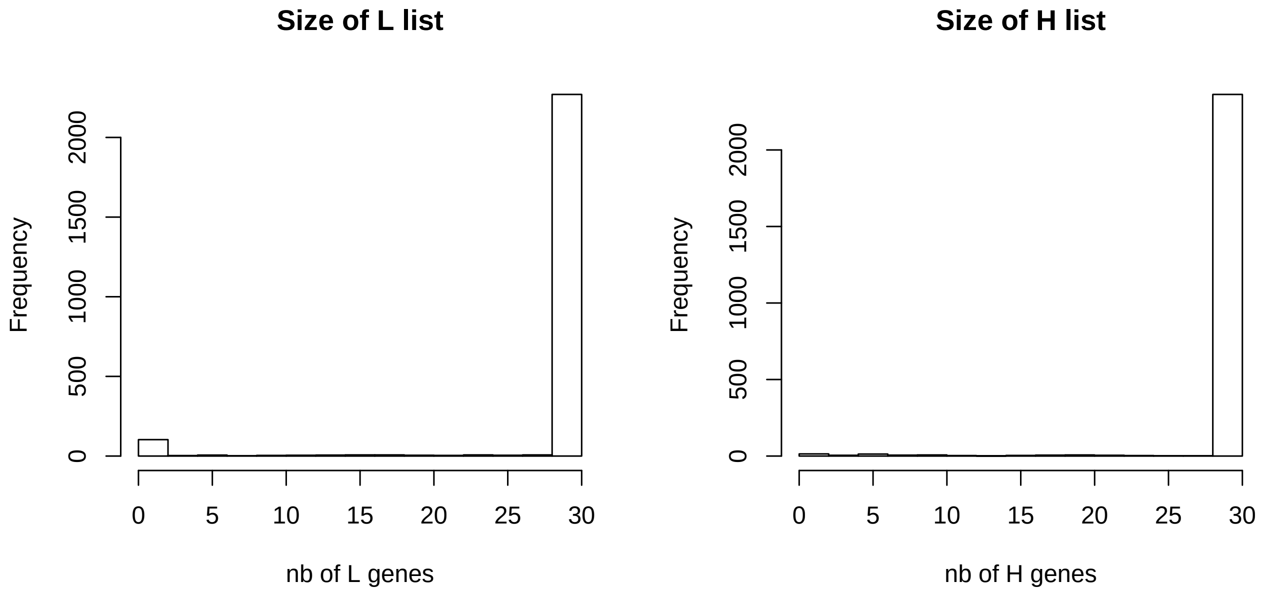

Differential expression testing

```r
threshold = 0.4
iterations = 20
quant_test = 0.05
factor_test = 1.2

penda_res = penda::penda_test(samples = data_case, controls = data_ctrl,
    threshold = threshold, iterations = iterations, L_H_list = L_H_list,
    quant_test = quant_test, factor_test = factor_test)
```

The function `penda_test` infers for each gene and for each sample of the `data_case` matrix its deregulation status (up-regulation, down-regulation or no deregulation). This function analyses case samples one by one. It is based on the `L_H_list` and tracks for changes in relative ordering in the sample of interest. If these changes exceed the given `threshold`, the gene of interest is considered as deregulated.

By default, the `threshold` parameter is set to 0.4 but we strongly advise users to use the vignette `vignette simulation` to adjust this parameter to the user-specific data.

Results are in the form of two matrices `$down_genes` and `$up_genes`. Each row corresponds to a gene and each column to a case sample. A TRUE entry in these matrices means that the corresponding genes are deregulated (down or up-regulated) in the corresponding samples.

```
#> Need help? Try Stackoverflow:
#> https://stackoverflow.com/tags/ggplot2.
```

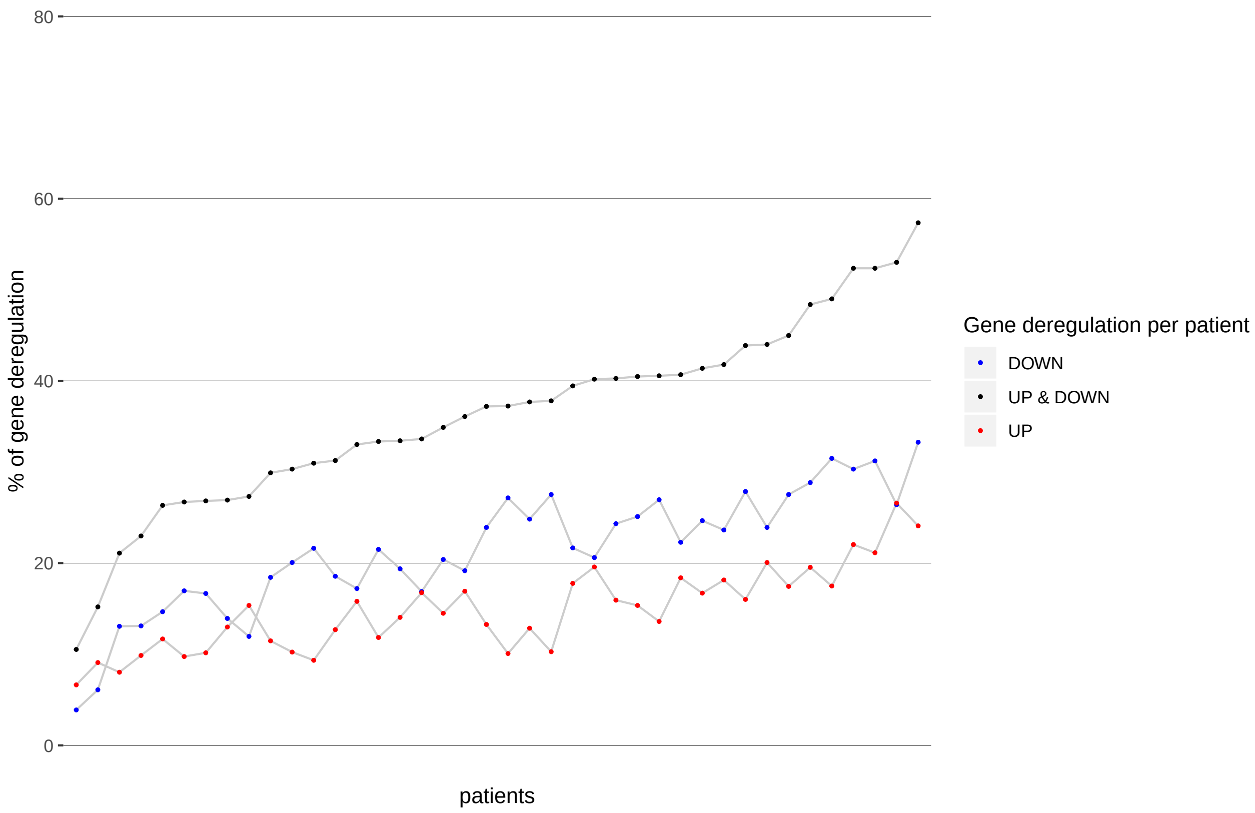
80
60
40
20
0
% of gene deregulation
patients
Gene deregulation per patient
DOWN
UP & DOWN
UP

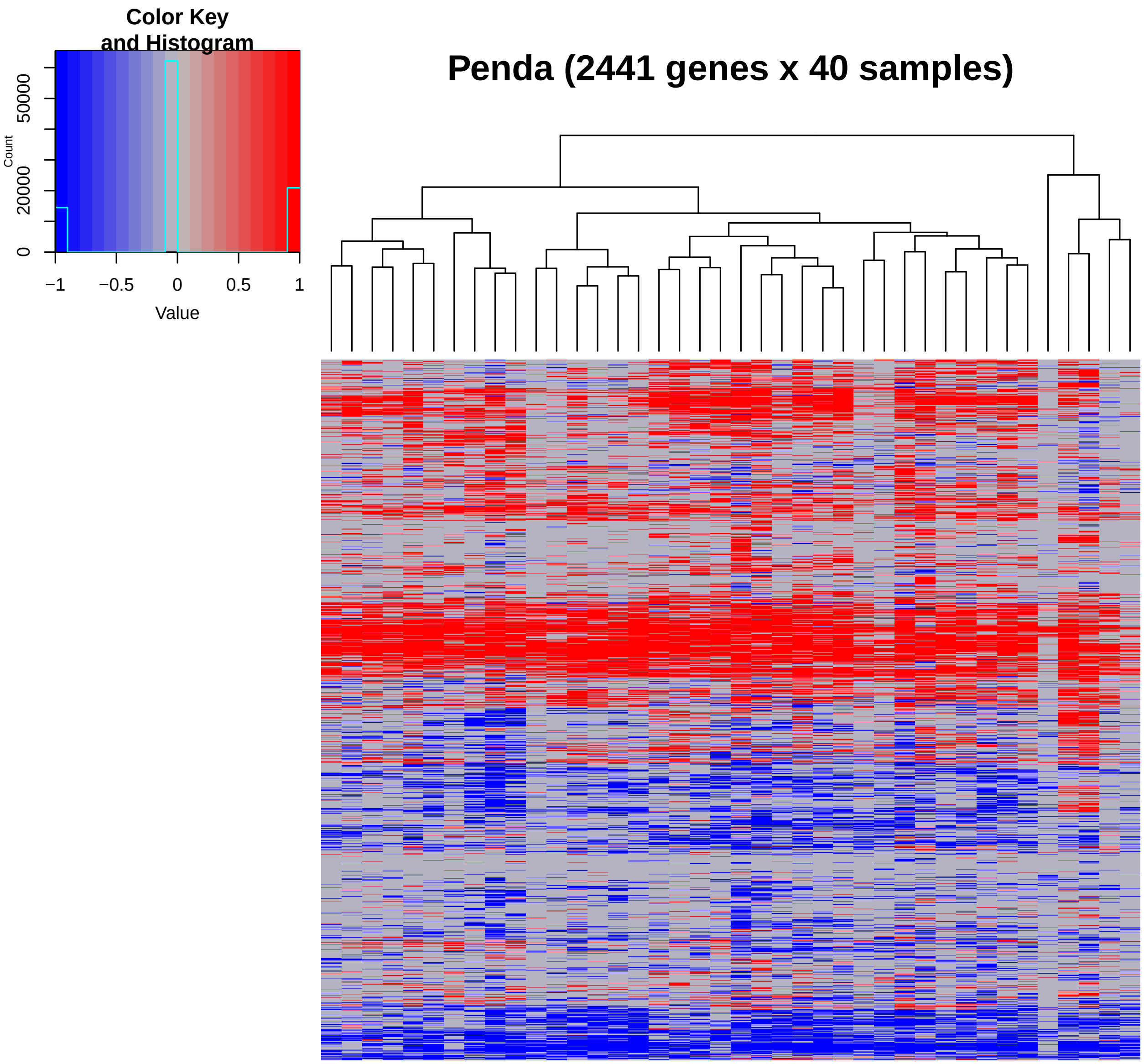

Material and methods

This paragraph is automatically generated by the vignette to specify the method and data filtering parameters. It can be directly cut and paste to the "material and methods" section of the user analysis.

The PenDA vignette of the `penda` package version 1.0 was executed on 3000 genes, using 40 control samples and 40 case samples.

The data set was pretreated as following: 0 genes and 0 samples were removed during the NA values filtering step, and 559 genes were removed because lowly expressed: under the threshold `val_min` = 483.92 in at least 99 % of cases.

40 controls were used to generate L and H lists using the following parameters: threshold LH = 0.99 and s_max = 30.

The PenDA method was then applied on 40 cases, with the following set of parameters: quantile = 0.05, factor = 1.2 and threshold = 0.4.

Session Information

```
sessionInfo()
#> R version 3.5.1 (2018-07-02)
#> Platform: x86_64-conda_cos6-linux-gnu (64-bit)
#> Running under: Debian GNU/Linux 8 (jessie)
#>
#> Matrix products: default
#> BLAS/LAPACK: /summer/epistorage/miniconda3/lib/R/lib/libRblas.so
#>
#> locale:
#>  [1] LC_CTYPE=en_US.UTF-8       LC_NUMERIC=C
#>  [3] LC_TIME=en_US.UTF-8        LC_COLLATE=en_US.UTF-8
#>  [5] LC_MONETARY=en_US.UTF-8    LC_MESSAGES=en_US.UTF-8
#>  [7] LC_PAPER=en_US.UTF-8       LC_NAME=C
#>  [9] LC_ADDRESS=C               LC_TELEPHONE=C
#> [11] LC_MEASUREMENT=en_US.UTF-8 LC_IDENTIFICATION=C
#>
#> attached base packages:
#> [1] stats     graphics  grDevices utils     datasets  methods   base
#>
#> other attached packages:
#> [1] ggplot2_3.1.1
#>
#> loaded via a namespace (and not attached):
#>  [1] Rcpp_1.0.1         penda_0.1.0        compiler_3.5.1
#>  [4] pillar_1.4.0       formatR_1.6        plyr_1.8.4
#>  [7] bitops_1.0-6       mixtools_1.1.0     tools_3.5.1
#> [10] digest_0.6.19      evaluate_0.13      tibble_2.1.1
#> [13] gtable_0.3.0       lattice_0.20-38    pkgconfig_2.0.2
#> [16] rlang_0.3.4        Matrix_1.2-17      yaml_2.2.0
#> [19] xfun_0.7           withr_2.1.2        stringr_1.4.0
#> [22] dplyr_0.8.1        knitr_1.22         caTools_1.17.1.2
#> [25] gtools_3.8.1       segmented_0.5-4.0  grid_3.5.1
#> [28] tidyselect_0.2.5   glue_1.3.1         R6_2.4.0
#> [31] survival_2.44-1.1  rmarkdown_1.12     gdata_2.18.0
#> [34] purrr_0.3.2        magrittr_1.5       gplots_3.0.1.1
#> [37] scales_1.0.0       htmltools_0.3.6    MASS_7.3-51.4
#> [40] splines_3.5.1      assertthat_0.2.1   colorspace_1.4-1
#> [43] labeling_0.3       KernSmooth_2.23-15 stringi_1.4.3
#> [46] lazyeval_0.2.2     munsell_0.5.0      crayon_1.3.4
```
