# Supplementary material for: PenDA, a rank-based method for personalized differential analysis: Application to lung cancer
Source: PLoS Comput Biol. 2020 May 11;16(5):e1007869. doi: 10.1371/journal.pcbi.1007869 (PMC7274464; doi:10.1371/journal.pcbi.1007869)
Supplement: S2 Text — (PDF) [file pcbi.1007869.s013.pdf]

PENDA: PErsoNalized Differential Analysis

Advanced User - Performing simulated personalized data analysis with `penda`

Magali Richard, Clementine Decamps, Florent Chuffart, Daniel Jost

2019-06-06

Introduction

`penda` (**PE**rsoNalized **D**ifferential **A**nalysis) is an open-access R package that detects gene deregulation in individual samples compared to a set of reference, control samples. This tutorial aims at providing to non-expert users basic informations and illustrations on how to run the package.

How to cite: Richard et al. (2019) PenDA, a rank-based method for Personalized Differential Analysis: application to lung cancer, in submission.

Dataset and data filtering

Dataset

The dataset used to illustrated the method corresponds to the transcriptomes of 3000 genes (RNAseq counts, normalized with DESeq2) for 40 normal, control samples and 40 tumorous samples taken from the TCGA study of lung adenocarcinoma [PMID:25079552].

`data_ctrl` is a data matrix containing the normalized counts of each control sample. The rownames of the matrix correspond to the gene_symbol, the colnames indicate the sample ID.

```
data_ctrl = penda::penda_data_ctrl
head(data_ctrl[,1:3])
#>       patient_55-6984-11 patient_43-6773-11 patient_55-6978-11
#> AADAC           347.2489           428.5498           442.0555
#> AAMP            965.2342          1528.3221           968.0266
#> ABCA1             0.0000             0.0000             0.0000
#> ABL1           1508.1784          1227.1325          1747.2431
#> ABL2            582.6719           645.4063           488.5088
#> ACACA             0.0000             0.0000             0.0000
dim(data_ctrl)
#> [1] 3000   40
```

`data_case` is a data matrix containing the normalized counts of each tumor sample. The rownames of the matrix correspond to the gene_symbol, the colnames indicate the sample ID.

```
data_case = penda::penda_data_case
data_case = data_case[rownames(data_ctrl),]
head(data_case[,1:3])
#>       patient_69-7764-01 patient_44-3919-01 patient_86-8278-01
#> AADAC           311.2129           374.9473          445.43169
#> AAMP           1466.5906           979.2256         1059.19225
#> ABCA1             0.0000             0.0000            0.00000
#> ABL1           2676.4306          2065.7474         2503.76905
#> ABL2           1167.0482           678.5603         1263.94317
#> ACACA             0.0000             0.0000           12.79693
dim(data_case)
#> [1] 3000   40
```

Note: this vignette is an example that has been designed for a rapid test of the method. So we limit the number of genes and the number of samples for this purpose. For an optimal utilization of the method, users should however upload all their available data (genes, control and case samples).

Extraction of data for simulations

The optimal choice of parameters (Sec. 4.2) is based on simulations that perturb control samples (Sec. 4.1). Here, we extract three patients (`data_simu`) from `data_ctrl` that will be used later for this purpose. For consistency, we also discard them from the `data_ctrl` matrix that will serve as reference.

```
data_simu = data_ctrl[,1:3]
data_ctrl = data_ctrl[,-(1:3)]
head(data_simu[,1:3])
#>       patient_55-6984-11 patient_43-6773-11 patient_55-6978-11
#> AADAC           347.2489           428.5498           442.0555
#> AAMP            965.2342          1528.3221           968.0266
#> ABCA1             0.0000             0.0000             0.0000
#> ABL1           1508.1784          1227.1325          1747.2431
#> ABL2            582.6719           645.4063           488.5088
#> ACACA             0.0000             0.0000             0.0000
dim(data_simu)
#> [1] 3000    3
dim(data_ctrl)
#> [1] 3000   37
```

Note: this vignette is an example that has been designed for a rapid test of the method. For a more complete analysis and a better parameter estimation, we recommend users to simulate more cases (10 for example instead of 3).

Data filtering

```
threshold_dataset = 0.99
Penda_dataset = penda::make_dataset(data_ctrl, data_case, detectlowvalue = TRUE,
    detectNA = TRUE, threshold = threshold_dataset)
#> [1] "0 probes are NA in at least 99 % of the samples."
#> [1] "0 patients have NA for at least 99 % of the probes."
#> [1] "Computing of the low threshold"
```

```
#> number of iterations= 97
#> [1] "159 genes have less than 23.1177736226348 counts in 99 % of the samples."
data_ctrl = Penda_dataset$data_ctrl
data_case = Penda_dataset$data_case
data_simu = data_simu[rownames(data_ctrl), ]
```

The function `make_dataset` contains three steps to prepare the data for the analysis.

- `detect_na_value` removes rows and columns (ie, genes and samples) of the data matrices that contain more than threshold % (default value = 0.99) of NA (Not Available) value.
- `detect_zero_value` removes genes with very low expression in the majority of samples (controls and cases), *ie.* genes whose expression is lower than `val_min` in `threshold`% of all the samples. By default it uses the function `normalmixEM` to estimate the value of `val_min` using all the *log2*-transformed count data but this parameter can also be tuned manually by the user.
- `rank_genes` sorts the genes based on the median value of gene expression in controls. This step is essential for the proper functioning of `penda`.

```
head(data_ctrl[,1:3])
#>         patient_77-8007-11 patient_55-6969-11 patient_22-4609-11
#> CAPZB            0.0000000           1.541098          0.8957864
#> CEACAM4          0.0000000           0.000000          0.0000000
#> DHX8             0.0000000           0.000000          0.0000000
#> DTNB             4.2227541           1.541098          0.0000000
#> EZH1             0.8445508           1.541098          0.0000000
#> GRIA1            0.0000000           0.000000          0.0000000
dim(data_ctrl)
#> [1] 2841   37
head(data_case[,1:3])
#>         patient_69-7764-01 patient_44-3919-01 patient_86-8278-01
#> CAPZB              1.29672          0.5895398          0.4921897
#> CEACAM4            0.00000          0.0000000          0.0000000
#> DHX8               0.00000          0.5895398          0.0000000
#> DTNB               0.00000          6.4849375          0.4921897
#> EZH1               0.00000          0.5895398         16.2422604
#> GRIA1              0.00000          0.5895398          0.0000000
dim(data_case)
#> [1] 2841   40
head(data_simu[,1:3])
#>         patient_55-6984-11 patient_43-6773-11 patient_55-6978-11
#> CAPZB             1.471394           0.000000           2.996986
#> CEACAM4           0.000000           0.000000           0.000000
#> DHX8              0.000000           0.000000           0.000000
#> DTNB              3.678484          10.326501           1.498493
#> EZH1              1.471394           3.442167           0.000000
#> GRIA1             0.000000           0.000000           0.000000
dim(data_simu)
#> [1] 2841    3
```

Relative gene ordering

```
threshold_LH = 0.99
s_max = 30
```

```
L_H_list = penda::compute_lower_and_higher_lists(data_ctrl, threshold = threshold_LH,
    s_max = s_max)
#> [1] "Computing genes with lower and higher expression"
L = L_H_list$L
H = L_H_list$H
```

The **penda** method uses the relative gene ordering in normal tissue.

The function `compute_lower_and_higher_lists` computes two matrices `L` and `H` based on the filtered control dataset (`data_ctrl`).

Each row of the **L** matrix contains a list of at most `s_max` (default value = 30) genes (characterized by their ids) whose expressions are **lower** than that of the gene associated to the corresponding row, in at least `threshold_LH` (default value = 99 %) of the control samples.

Each row of the **H** matrix contains a list of at most `s_max` (default value = 30) genes (characterized by their ids) whose expressions are **higher** than that of the gene associated to the corresponding row, in at least `threshold_LH` (default value = 99 %) of the control samples.

Below, for some genes (FOXH1, KRTAP2-3, etc.), we show the id of 10 genes of the L and H lists.

```
L[1000:1005,1:10]
#>          [,1] [,2] [,3] [,4] [,5] [,6] [,7] [,8] [,9] [,10]
#> DDOST     783  705  685  684  677  675  660  652  649   641
#> SMCO1     776  768  760  753  742  740  733  725  721   715
#> KRTAP2-3  868  864  851  849  847  828  816  813  809   804
#> SIGLEC16  813  804  798  797  788  787  770  763  753   746
#> KIAA0895L 827  815  804  798  796  791  788  787  785   782
#> IQSEC1    857  807  799  782  777  775  768  764  763   760
H[1000:1005,1:10]
#>          [,1] [,2] [,3] [,4] [,5] [,6] [,7] [,8] [,9] [,10]
#> DDOST    1300 1485 1520 1576 1615 1756 1767 1809 1889  1905
#> SMCO1    1304 1336 1343 1346 1349 1351 1355 1363 1368  1376
#> KRTAP2-3 1180 1181 1189 1195 1198 1200 1202 1203 1214  1218
#> SIGLEC16 1271 1282 1319 1323 1328 1332 1333 1334 1339  1343
#> KIAA0895L 1329 1398 1460 1495 1498 1508 1522 1523 1525  1526
#> IQSEC1   1223 1248 1306 1309 1320 1328 1338 1359 1361  1366
dim(L)
#> [1] 2841   30
dim(H)
#> [1] 2841   30
```

Define optimal parameters from simulations

Generation of the simulated dataset

Estimation of optimal parameters adapted to the user data is based on a ROC analysis on simulated datasets. The function `complex_simulation` uses the real distribution of difference between the control and the case samples to simulate the proportion and the value of the dysregulation (see the original paper for details on the method of simulation).

It returns the vector of initial data (`data_simu`, in `simulation$initial_data`), the vector of data with modifications (`simulation$simulated_data`) and the index of modified data (`simulation$changes_idx`).

```r
size_grp = 100
quant_simu = 0.05
simulation = penda::complex_simulation(data_ctrl, data_case,
    data_simu, size_grp, quant = quant_simu)
#> [1] "Computing genes groups"
#> [1] "Simulating dysregulation of 3 patients."
head(simulation$initial_data)
#>         patient_55-6984-11 patient_43-6773-11 patient_55-6978-11
#> CAPZB             1.471394           0.000000           2.996986
#> CEACAM4           0.000000           0.000000           0.000000
#> DHX8              0.000000           0.000000           0.000000
#> DTNB              3.678484          10.326501           1.498493
#> EZH1              1.471394           3.442167           0.000000
#> GRIA1             0.000000           0.000000           0.000000
head(simulation$simulated_data)
#>         patient_55-6984-11 patient_43-6773-11 patient_55-6978-11
#> CAPZB             1.471394           0.000000          81.700433
#> CEACAM4         112.867321           0.000000          15.425735
#> DHX8              0.000000           0.000000           0.000000
#> DTNB              3.678484          10.326501           1.498493
#> EZH1              1.471394           3.442167          15.084564
#> GRIA1             0.000000           0.000000           0.000000
```

```r
simulation$changes_idx[1000:1005]
#> [1] 3528 3533 3534 3535 3537 3538
#Before simulation:
simulation$initial_data[simulation$changes_idx[1000:1005]]
#> [1] 332.16910 154.89751 521.48828 103.26501  99.82284 103.26501
#After simulation:
simulation$simulated_data[simulation$changes_idx[1000:1005]]
#> [1]  858.020717   20.398067 1246.969691  973.211691   12.512462    9.348681
```

Optimal parameter choice

For the quantile method

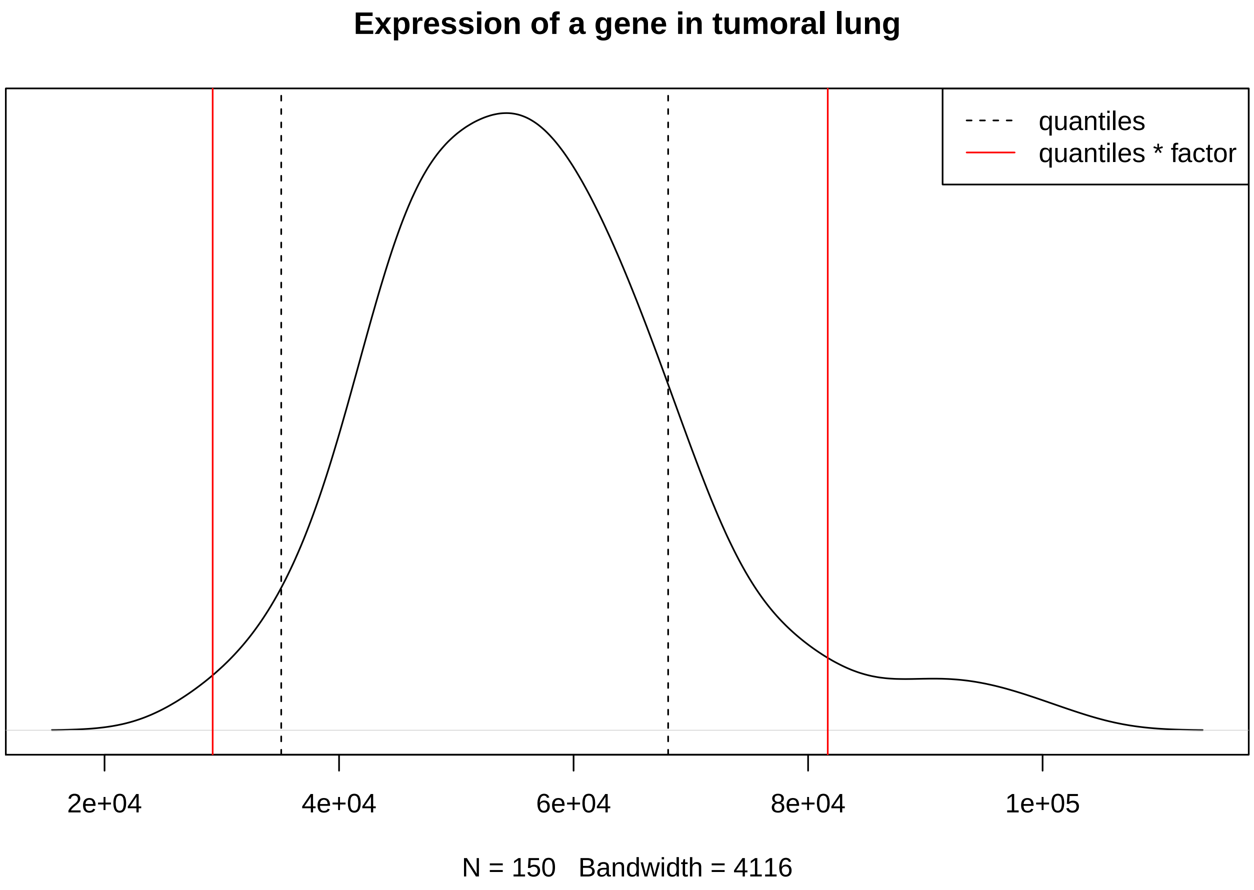

In the rare cases where the lists L or H of a gene are empty, `penda` uses a simpler, less efficient, method based on quantile to determine the deregulation status (see the original paper). This quantile method depends on two parameters:

- the `quantile` values of the distribution of expression of a gene in the control samples (dotted line)
- the `factor` parameter which modulates the quantile values and defined the thresholds which determine the deregulation status in case samples (red line).

The function `choose_quantile` applies the quantile method to the simulated dataset for various values of the parameters quantile and factor (grid of values defined by the variables `factor_values` and `quantile_values`, see the R documentation for default parameters). For each set of parameters, it computes the corresponding False Discovery Rate (FDR), True Positive Rate (TPR) and False Positive Rate (FPR). The function `select_quantile_param` then choose the best set that maximize the TPR for a user-specified maximal value of the FDR (defined by the variable `FDR_max`, default value = 0.15).

```
quantile_values = c(0, 0.01, 0.02, 0.03, 0.05, 0.06, 0.08, 0.1,
    0.15, 0.2, 0.3, 0.4)
factor_values = c(1, 1.2, 1.4)
which_quantile = penda::choose_quantile(data_ctrl, simulation,
    factor_values = factor_values, quantile_values = quantile_values)
best_quantile = penda::select_quantile_param(which_quantile,
    FDR_max = 0.1)
```

In this example, optimum quantile method parameters are defined as:

- quantile = 0.05
- factor = 1.2

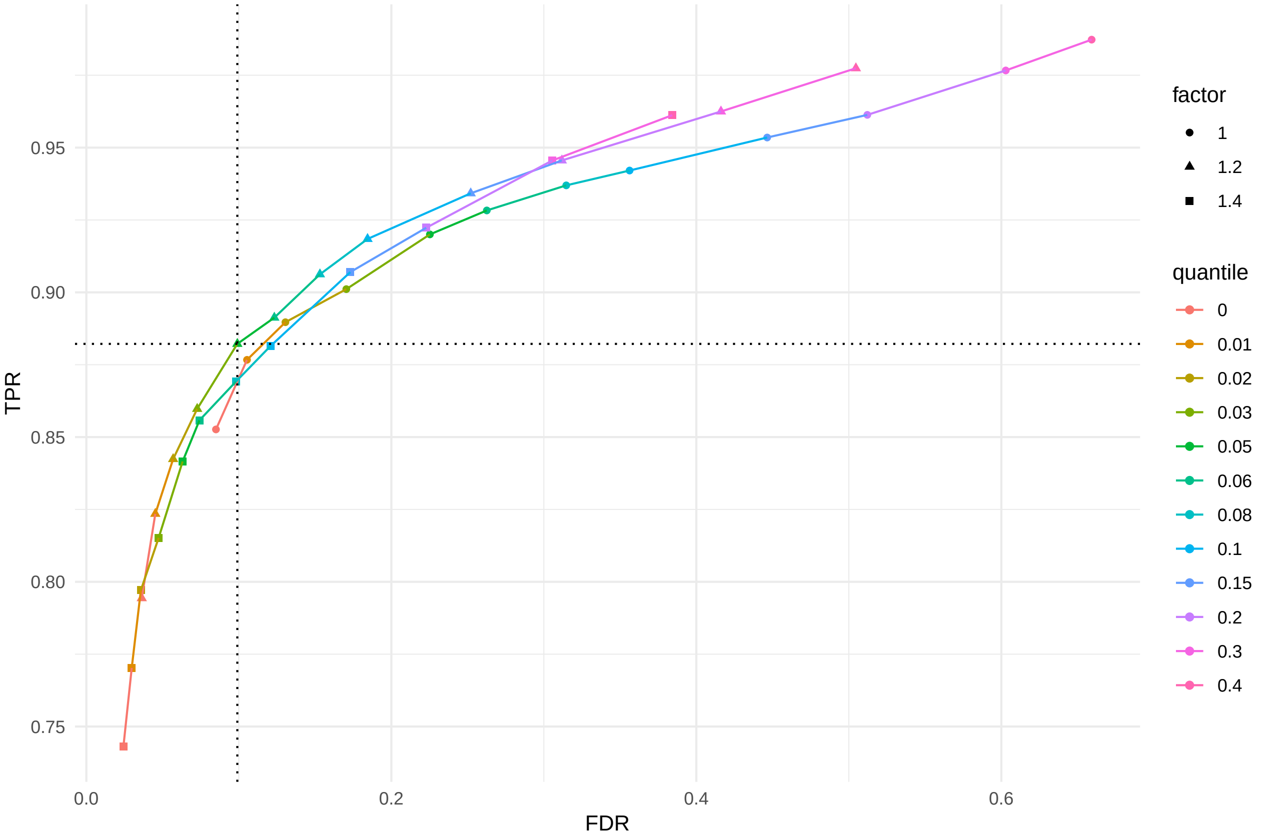

Note: this vignette is an example that has been designed for a rapid test of the method. For a more complete analysis and a better parameter estimation, we recommend users to simulate more cases (10 for example instead of 3) and test more values for the parameters quantile and factor.

For the PenDA method

The `penda` method infers fo each gene in each case sample its deregulation status (up-regulation, down-regulation or no deregulation) based on the `L_H_list`. It tracks for changes in relative ordering in the sample of interest. If these changes exceed the given threshold, the gene of interest is considered as deregulated.

The second step of the parameter choice is therefore to determine the optimal value of `threshold`. The function `choose_threshold` applies PenDA to the simulated dataset for different threshold values (defined by the variable `threshold_values`) and computes the corresponding FDR, TPR and FPR. The function `select_threshold_param` then choose the threshold value that maximize the TPR for a user-specified maximal value of the FDR (defined by the variable `FDR_max`, default value = 0.05).

```
threshold_values = c(0, 0.05, 0.1, 0.3, 0.4, 0.5, 0.6, 0.7, 0.8,
    0.9)
best_quant = best_quantile$quantile
best_fact = best_quantile$factor
which_threshold = penda::choose_threshold(data_ctrl, L_H_list,
    30, simulation, threshold_values, quant_test = best_quant,
    factor_test = best_fact)
```

```
best_threshold = penda::select_threshold_param(which_threshold,
    FDR_max = 0.05)
```

In this example, optimum test threshold parameter is defined as:

- threshold = 0.4

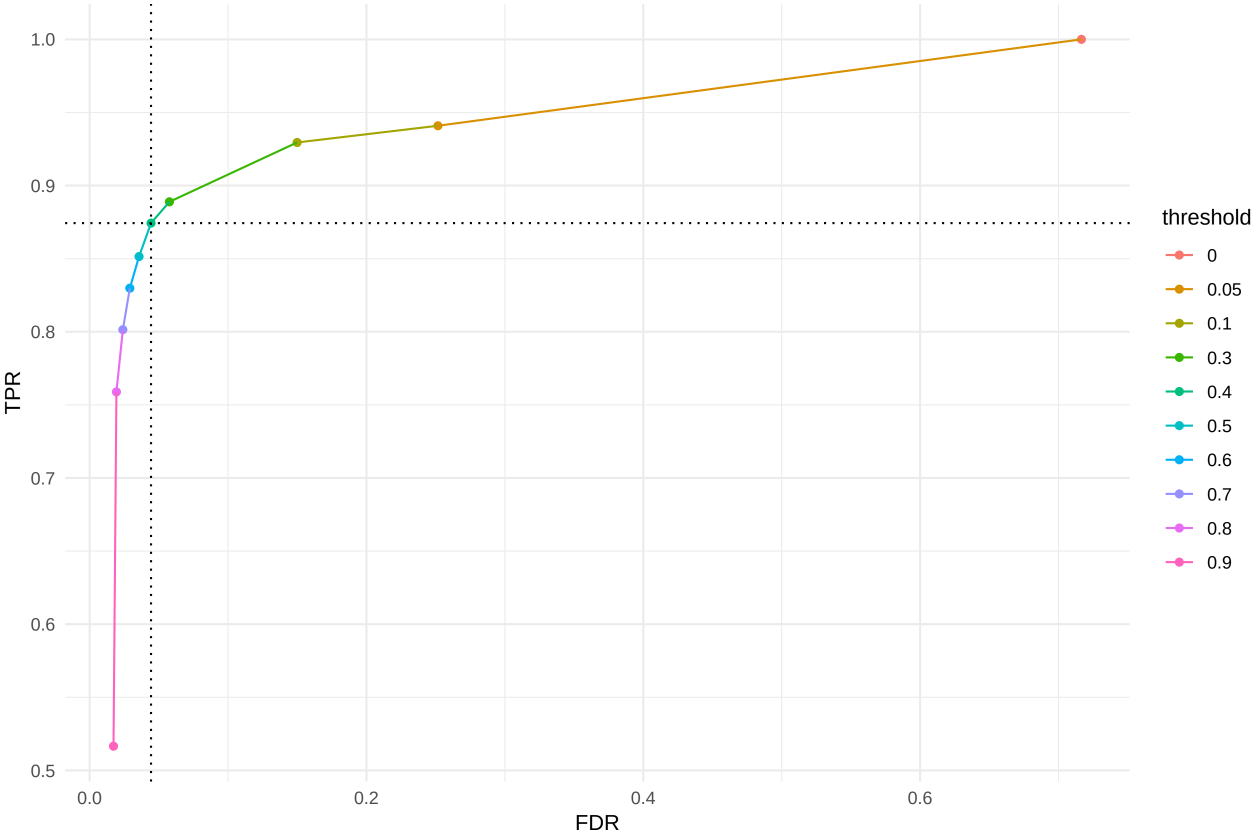

Note: this vignette is an example that has been designed for a rapid test of the method. For a more complete analysis and a better parameter estimation, we recommend users to simulate more cases (10 for example instead of 3) and test more values for the parameter threshold.

Test for false positive in control samples

As a safety check, PenDA is applied to the control samples used for the simulations and estimates the proportion of false positives.

```
threshold_values = c(0, 0.05, 0.1, 0.3, 0.4, 0.5, 0.6, 0.7, 0.8,
    0.9)

best_quant = best_quantile$quantile
best_fact = best_quantile$factor
results = c()
for (thres in threshold_values) {
    penda_res = penda::penda_test(samples = data_simu, controls = controls,
        threshold = thres, iterations = 20, L_H_list = L_H_list,
        quant_test = best_quant, factor_test = best_fact)
    results = rbind(results, c("U", thres, colSums(penda_res$up_genes)))
```

```
    results = rbind(results, c("D", thres, colSums(penda_res$down_genes)))
}
```

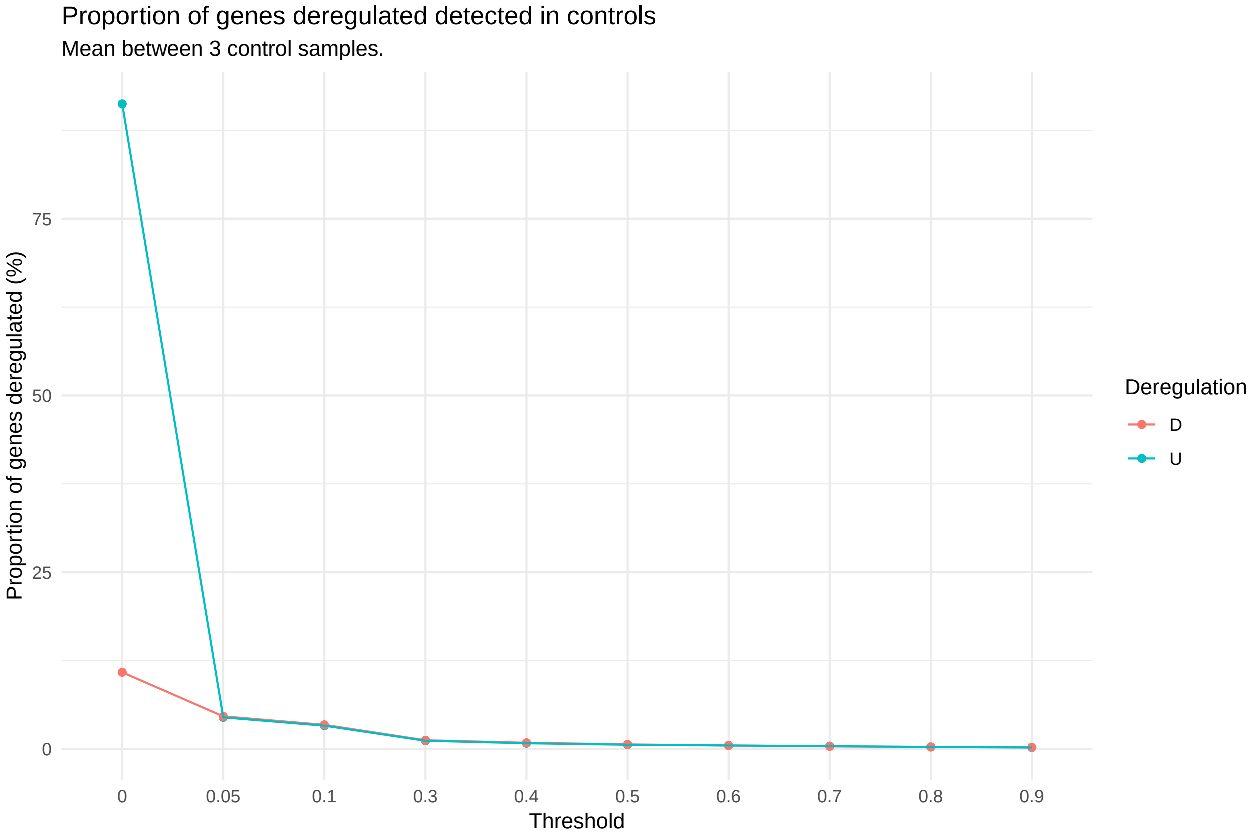

Summary of simulation results

With these simulations you can now perform analysis on your real data (see `vignette_penda`) using the parameters:

- quantile = 0.05
- factor = 1.2
- threshold = 0.4

Material and methods

This paragraph is automatically generated by the vignette to specify the method and data filtering parameters. It can be directly cut and paste to the "material and methods" section of the user analysis.

The simulation vignette of the `penda` package version 1.0 was executed on 3000 genes, using 37 control samples and 40 case samples.

The data set was pretreated as following: 0 genes and 0 samples were removed during the NA values filtering step, and 159 genes were removed because lowly expressed: under the threshold `val_min` = 23.12 in at least 99 % of cases.

3 cases samples were simulated using the complex simulation function with the following parameters: group size = 100, quantile = 0.05. Theses simulations identified 29.8% of genes as typically deregulated in cases

samples.

37 controls were used to generate L and H lists using the following parameters: threshold LH = 0.99 and s_max = 30.

The quantile method was applied on the 3 simulated cases. We retained a global FDR value of 0.099, with the following set of parameters: quantile = 0.05 and factor = 1.2.

The PenDA method was then applied on these 3 cases. We retained a global FDR value of 0.0444 , with the following set of parameters: quantile = 0.05, factor = 1.2 and threshold = 0.4.

Session Information

```
sessionInfo()
#> R version 3.5.1 (2018-07-02)
#> Platform: x86_64-conda_cos6-linux-gnu (64-bit)
#> Running under: Debian GNU/Linux 8 (jessie)
#>
#> Matrix products: default
#> BLAS/LAPACK: /summer/epistorage/miniconda3/lib/R/lib/libRblas.so
#>
#> locale:
#>  [1] LC_CTYPE=en_US.UTF-8       LC_NUMERIC=C
#>  [3] LC_TIME=en_US.UTF-8        LC_COLLATE=en_US.UTF-8
#>  [5] LC_MONETARY=en_US.UTF-8    LC_MESSAGES=en_US.UTF-8
#>  [7] LC_PAPER=en_US.UTF-8       LC_NAME=C
#>  [9] LC_ADDRESS=C               LC_TELEPHONE=C
#> [11] LC_MEASUREMENT=en_US.UTF-8 LC_IDENTIFICATION=C
#>
#> attached base packages:
#> [1] stats     graphics  grDevices utils     datasets  methods   base
#>
#> other attached packages:
#> [1] ggplot2_3.1.1
#>
#> loaded via a namespace (and not attached):
#>  [1] Rcpp_1.0.1         penda_0.1.0        compiler_3.5.1
#>  [4] pillar_1.4.0       formatR_1.6        plyr_1.8.4
#>  [7] mixtools_1.1.0     tools_3.5.1        digest_0.6.19
#> [10] evaluate_0.13      tibble_2.1.1       gtable_0.3.0
#> [13] lattice_0.20-38    pkgconfig_2.0.2    rlang_0.3.4
#> [16] Matrix_1.2-17      yaml_2.2.0         xfun_0.7
#> [19] withr_2.1.2        stringr_1.4.0      dplyr_0.8.1
#> [22] knitr_1.22         segmented_0.5-4.0 grid_3.5.1
#> [25] tidyselect_0.2.5   glue_1.3.1         R6_2.4.0
#> [28] survival_2.44-1.1 rmarkdown_1.12     purrr_0.3.2
#> [31] magrittr_1.5       scales_1.0.0       htmltools_0.3.6
#> [34] MASS_7.3-51.4      splines_3.5.1      assertthat_0.2.1
#> [37] colorspace_1.4-1   labeling_0.3       stringi_1.4.3
#> [40] lazyeval_0.2.2     munsell_0.5.0      crayon_1.3.4
```
