# Supplementary material for: PenDA, a rank-based method for personalized differential analysis: Application to lung cancer
Source: PLoS Comput Biol. 2020 May 11;16(5):e1007869. doi: 10.1371/journal.pcbi.1007869 (PMC7274464; doi:10.1371/journal.pcbi.1007869)
Supplement: Attachment — Submitted filename: rebuttal.docx [file pcbi.1007869.s018.docx]

Dear Editor,

Thank you for giving us the opportunity to revise our manuscript according to reviewers’ minor recommendations.

We would like to acknowledge the reviewers for their very positive comments and their insightful suggestions. We have now revised the manuscript accordingly (see our point-by-point responses below).

Thank you again for your consideration, and we are looking forward to publish our manuscript in PLoS Computational Biology.

Sincerely yours

Magali Richard and Daniel Jost

*Reviewer #1: The authors presented a unique approach for identifying dysregulated genes in cancer data. The overall quality of the study, including writing, organization, and presentation is good. I wasn’t familiar with this type of approach, this manuscript does make a good introduction and I found it has the potential to be useful in future research studies.*

**We thank the reviewer for his/her enthusiasm.**

*Some minor comments*

*1. The authors summarized DEseq2 edgeR, limma to be fold-change method. These methods do provide fold change, but their algorithm is much more than just fold change.*

**We agree that DESeq2, edgeR & limma are more than fold-change methods. We slightly modified the sentence to avoid confusion.**

*2. Detail of how data was simulated should be provided.*

**The full details on the simulation method are given in the Methods section ‘Simulated datasets’. In the Results section ‘Impact of method parameters and of the dataset properties on performance’, we already have a small paragraph describing it briefly. We added another sentence in this paragraph to detail it a bit more.**

*3. Small grammar errors such as “Help to found”*

**We made a careful reading of the manuscript to avoid typo and grammar errors.**

*Reviewer #2: In this interesting manuscript, Richard et al. introduce a new method, PenDA, for identifying genes that are deregulated in individual samples, based on comparison to a reference group of samples. The method works by comparing the ranking of each query gene relative to a set of genes that are consistently above or below the query gene in the reference group, and whose overall expression level is as close to the query gene as possible. The method is very simple, but in my opinion the authors have adequately shown that despite its simplicity, it performs quite well. The performance evaluation is almost entirely based on simulated data, but the simulations are performed carefully to take into account differences in behaviour of genes with varying baseline expression levels, and also to study the effect of various parameters that can be potentially tuned. The authors have then applied their method to TCGA lung cancer data, showing that their approach can identify differences between tumour and normal, can distinguish lung adenocarcinoma from squamous cell lung carcinoma, and can further identify potentially new subtypes within the adenocarcinoma cohort that correlate with genomic subtypes. Overall, I enjoyed reading the manuscript, and have only minor comments with respect to some of the details of the method evaluation and comparison:*

**We thank the reviewer for his/her very positive evaluation of our work.**

*1. In their simulations, the authors randomly choose a fraction of genes as deregulated, and modify the expression of these genes in a way that the amount of change in gene expression exceeds the natural variation that is seen in control (normal) samples (< 5th percentile or > 95th percentile). Does the choice of the percentile threshold affect the results? Can the authors perform simulations with at least one other percentile threshold (e.g. < 20th and > 80th percentiles) to show that the method performance is robust with respect to how the simulated data are constructed? Or if the performance is decreased, does the same apply to other methods that PenDA is compared to?*

**We tested the performance of PenDa when varying simulation parameters (Additional File 1: Figure S11a,b). We observed that the choice of the packet size does not affect the results Figure S11a). For the percentiles (Figure S11b), since the choice of the percentiles will impact the proportion of deregulated genes and how a gene expression may be considered as ‘normal’, we expected that it may impact the TPR/FPR analysis. Indeed, for example in the extreme case suggested by the reviewer (20%,80%: meaning that ~40% of the difference in gene expression between normal samples may be considered as deregulated), we observed an increase in the proportion of deregulated genes (~60%) and the performance of PenDa are lower (still reaching 70% TPR at 5% FPR). However, PenDA still outperforms the other methods (whose performances are also impacted), in particular in the low FPR range.**

**We added a sentence in the Methods section dedicated to simulation to mention this point and to refer to the new FigS11a,b.**

*2. Among several different methods that the authors have evaluated, they test the ability of DESeq2 to identify deregulated genes in simulated data. My understanding from the Methods section is that the simulations are performed based on normalized data, and therefore the simulated dataset will also on the same normalized scale. However, DESeq2 requires raw read counts. Could the authors please clarify?*

**DESeq2 does not require *raw* read counts per se to perform differential analysis. It needs counts. To correct for library size and possible batch effects (that certainly are more present in raw read counts), prior to differential analysis, DESeq2 normalizes data based on the “median ratio method”. In our case, with our already normalized simulated datasets, skipping the normalization step in DESeq2 does not impact significantly the predictions (Additional File: Figure S11c). However, if we use non-standardized simulated data (as in Figure 3c), skipping the normalization effect dramatically affects the performance of the method (Additional File: Figure S11c). We added sentences in the Results section dedicated to comparing PenDA with other methods and in the Methods section dedicated to DESeq2 to mention this point and to refer to the new FigS11c.**

*3. The authors show that the lung adenocarcinoma samples can be divided into three subtypes (classes I, II, and III) based on PenDA differential expression status. They state in the manuscript that a previous work could not identify these classes based on normalized counts, but it is not clear to me if this really shows the effect of PenDA vs. normalized counts, or if it reflects other potential differences in the analysis approach. Can the authors please repeat the same hierarchical clustering analysis using the same genes but with normalized counts, and compare to PenDA-based clustering?*

**We performed a similar hierarchical clustering analysis (HCA) on the normalized counts (Additional file 1 : Fig S8 B). While HCA on normalized counts separates ADC and SQCC tumors almost as nicely as HCA on PenDA results (as for the principal component analysis, Fig.6a-c), it is less clear that the different subclasses for ADC found with PenDA are also captured by the HCA on normalized counts (see new Fig S8B). For example, by cutting the resulting tree into 4 clusters, PenDA-based ADC subclass II is split into 3 groups of significant sizes. Similarly, 2 clusters contain a mixture of PenDa-based ADC subclasses.**

**We added a sentence in the text to mention this point and to refer to the new Fig S8B.**
